# The effect of replacing dental needles between subsequent inferior alveolar nerve blocks on pain and trismus – a double blinded, randomised split-mouth study

**DOI:** 10.4317/jced.61784

**Published:** 2025-01-01

**Authors:** Tony Skapetis, Adelewa Olakitan Idowu, Thiruni Anushka Fernando, Gillian Marvel, Kanisha Vrajesh Mehta, Herdeza Verzosa

**Affiliations:** 1 The University of Sydney School of Dentistry, Faculty of Medicine and Health, The University of Sydney, Sydney, NSW, Australia; 2 NSW Health, Western Sydney Local Health District, Westmead, NSW, Australia

## Abstract

**Background:**

Inferior alveolar Nerve Blocks are widely used in dental practice for achieving anaesthesia in the mandibular teeth. It is widely accepted that in order for this type of injection to be effective, the needle needs to penetrate a substantial depth of soft tissues as well as make contact with bone. This routinely leads to both blunting and barbing of the needle tip suggesting that a needle change is preferable for any subsequent injection as this will result in less tissue damage and pain for patient’s. The study aimed to verify whether a change in needle affected measures of pain and trismus.

**Material and Methods:**

This was a prospective, single center, double blinded (both clinician and participant), randomized, split-mouth study conducted in a large dental hospital and teaching facility. Participants were screened for factors that might alter their head pain sensation and participated in 2 clinical visits. In the first visit 2 needle insertions either with/without needle change were performed without anaesthetic as per an IANB injection. A survey instrument was used to capture several dimensions of pain both immediately after the visit as well as 48-72 hours later. This method was repeated on a second visit on the opposite mandibular quadrant after a washout period of at least 2 weeks. Paired t-Test’s were performed at the 2 time points.

**Results:**

Significance was only demonstrated in one of 10 sensory and 12 emotional pain descriptors during one of the 2 time points of measurement. Similarly, VAS mean pain scores and a measure of trismus were not affected by needle change.

**Conclusions:**

This study was able to demonstrate that a change in needle between subsequent IANB’s does not affect self-reported measures of pain nor trismus.

** Key words:**Nerve block, dental injection, dental anaesthesia, pain, trismus.

## Introduction

The inferior alveolar nerve block (IANB) technique is commonly used prior to dental procedures to anaesthetise the mandibular posterior teeth prior to dental procedures. It involves inserting either a 25- or 27-gauge needle into the pterygomandibular space to access the inferior alveolar nerve, which supplies the lower teeth (1). With respect to this technique, the needle tip must pass through several soft tissues layers as well as contact bone (2).

Electron microscopy studies have shown that needle tips are significantly deformed and/or blunted following penetration of the oral mucosa soft tissue, as well as following bone contact (3,4). During the administration of an IANB, if adequate anaesthesia is not achieved with the first insertion then a repeat injection is normally given with the now blunted needle thereby potentially causing additional soft tissue trauma (4); this has the potential of causing greater patient pain as well as an increased risk of postoperative complications, such as trismus (5).

Despite previous research demonstrating needle tip deformation after single use (4,5) and potential for improvements in patient comfort (6), it is not common practice to replace the dental needle for repeat IANBs for the same patient (2).

Several randomized control trials (RCT) have been conducted surrounding IANB’s such as those relating efficacy relative to injection speed (7) and needle length and gauge (8) as well as a systematic review and meta-analysis of RCT’s comparing various local anaesthetic solutions to IANB success (9). However, a gap in the literature was identified with no randomized clinical studies that have compared the difference between replacing and not replacing the dental needle between repeat IANB injections in terms of post-operative pain experience and/or trismus.

This study’s primary outcome was to either verify or reject the null hypothesis applied to the measurement of pain and trismus following a second IANB injection, with and without a needle change. Additionally, the study’s secondary outcome was to verify whether needle tip deformation following bone contact, could clinically justify needle change between subsequent IANBs, as was previously suggested in the literature.

## Material and Methods

-Study design and setting.

**Figure 1:**
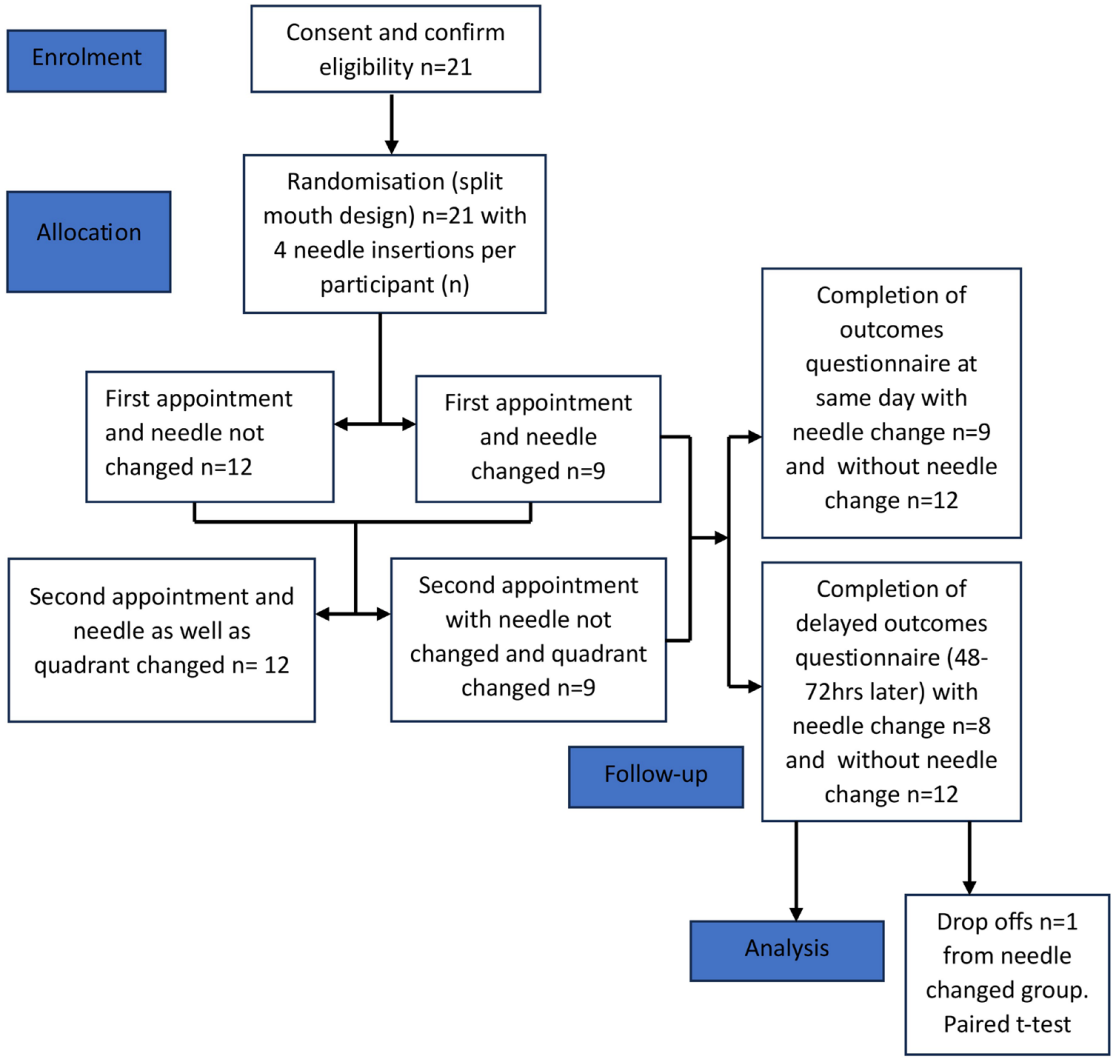
CONSORT-2010-Checklist (1).

This was a prospective, single center, double blinded (both clinician and participant), randomized and self-controlled, split-mouth study conducted at Westmead Center for Oral Health (WCOH), Sydney, Australia during the 2022 calendar year. The research has been approved by Western Sydney Local Heath District (WSLHD) Human Research Ethics Committee; ref: 2020/ETH03019.The study was ceased at the end of 2022 when adequate recruitment numbers were achieved. The study protocol is summarized in Figure 1 as per CONSORT guidelines and checklist included.

-Participants and recruitment:

Invitations to voluntarily participate in the study were distributed using flyers and Facebook year group posts among educators and students of the University of Sydney Dental School (USDS). The flyers/posts contained a hyperlink to an online recruitment page contained within the application REDCap which was used to securely manage the initial consent, screening questionnaire for determining eligibility and pain survey instrument. Verbal and written consent was additionally obtained at the start of the first clinical visit by one of two senior clinicians with one of them undertaking over 90% of the needle insertions. The 2 clinicians extensively discussed their technique prior to commencement to maintain uniformity and calibration.

-Inclusion and exclusion criteria:

Persons 18 years of age and above, proficient in English with the ability to attend the two clinical visits were invited to enroll.

All interested participants completed a screening questionnaire to exclude any odontogenic or non-odontogenic disease process, systemic condition or medications that may influence pain response. Additionally, those with a history of bleeding disorders, dental anxiety and needle phobia causing fainting were also excluded from participation. Given that all participants were well educated and could understand the nature of this research, self-exclusion was possible during the initial completion of the screening questionnaire.

-Randomisation and concealment:

Randomly generated treatment allocations were placed within sealed opaque windowed envelopes by a research assistant and used to assign participants to one of four allocations at the first clinical visit. Both participant and clinician were blinded to the assignment and allocation of one of four groups:-

a) first insertion quadrant 3: without needle change between a subsequent IANB injection in the same quadrant and at the same clinical visit.

b) first insertion quadrant 3: with needle change between a subsequent IANB injection in the same quadrant and at the same clinical visit.

c) first insertion quadrant 4: without needle change between a subsequent IANB injection in the same quadrant and at the same clinical visit.

d) first insertion quadrant 4: with needle change between a subsequent IANB injection in the same quadrant and at the same clinical visit.

-Clinical Protocols:

Initial (1st) appointment.

Participants were positioned upright, in the dental chair and a research assistant would pass the clinician a dental syringe attached to a 27G, 38mm needle loaded with a 2.2ml anaesthetic cartridge (reversed within the barrel of the syringe so that no solution could be injected). The needle type/brand was kept constant throughout the study. Both clinician and study participant would be then advised which quadrant the first needle insertion was to take place using a direct IANB technique into the pterygotemporal depression(2). After bony contact and without aspiration, the needle/syringe was withdrawn from the mouth, recapped and placed into a safe container and removed from the clinic. The needle was subsequently either changed (with the seal broken) or not changed depending on the randomization and returned to the clinician 3 minutes later. A second insertion would then take place in the same quadrant after which the participant would be escorted to another room to complete an online outcomes survey on an iPad in privacy. The participant was then instructed to provide a delayed survey response by re-doing the same survey 48-72 hours after the initial appointment with a reminder sent.

-Follow-up (2nd) appointment.

A washout period of at least 2 weeks after the initial appointment was allocated, to allow any affects from the 1st visit to resolve. An identical protocol with 2 needle insertions was conducted except for a change in both the mandibular quadrant, as well as either a change or not of the needle. The latter was dependent on what had occurred in the 1st visit, while maintaining the blinding of clinician and study participant. Additionally, same day (immediate) and delayed online survey responses were obtained.

-Instruments used to measure outcomes.

A self-administered (same day and follow-up) survey was constructed from previously validated instruments which consisted of 4 sections:- 

1) Visual analogue scale (VAS) measuring pain from no pain to worst possible pain on a 0-100mm, 10 unit linear scale.

2) A 10 question sensory pain measure, using a 4 point Likert Scale with responses ranging from none to mild, moderate and severe.

3) An 12 question emotional pain measure, using a 6 point Likert scale with severity scores ranging from 0-5.

4) A single question relating to the degree of mouth opening and by inference, the presence/absence of trismus, by asking if the participant was able to fit the 3 middle fingers vertically between their upper and lower central incisors.

-Power calculations.

A power calculation was performed using G*Power software (6). The clinically relevant difference in the VAS score was set to 2 units. A minimum sample of 20 subjects (dental quadrants) per study arm was required to detect statistical significance with 80% power applying a two-tailed significance level of α=0.05 and allowing for a 20% attrition rate.

-Statistical analysis.

Frequencies together with the paired samples t-tests was performed between the 2 groups of data, namely with and without a needle change using the same day scores ( i.e. scores obtained immediately after needle insertions in clinical visits 1 and 2) and the second group consisting of the 2 delayed scores ( 48-72hrs after each of the clinical visits). Significance was set at *p*≤0.05 within a 95% confidence interval as calculated using SPSS® software.

## Results

Following randomisation, 12 participants were allocated to the no needle change group and 9 to the changed needle group, during their first clinical appointment.

-Same day scores:

Twenty-one participants, one of which was an educator with the remainder, students, were recruited into the study with all completing the same day scores. VASs’ for pain ranged between 4-73 and 10-73 for no needle change (with a mean of 33.05) and changed needle (with a mean of 38.62) groups respectively.

 Of the 10 questions relating to descriptors of sensory pain, the descriptor “sharp” gave the highest mean scores of x = 2.05 ( no needle change) and x =2.52 (needle changed) and “splitting’ the lowest mean scores (x=1.0 for no change and x =1.14 needle changed). Using the single and 2-tailed paired samples t-test, only the descriptor “sharp” (pair 4) showed significance with a value of *p*=0.009 and a higher mean pain score with no needle change (x =2.52) and a confidence interval of 95% ( Table 1). Similarly, no significance was demonstrated in the paired VASs’.

In terms of emotional pain, no significance was noted when comparing the paired scores across all the 12 descriptors with and without a needle change. The descriptors; guilty, powerless, confidence, recognize and confusion were omitted from  Table 1, as their means were identical with and without needle change (NoCh-Ch) and t could not be computed. Additionally, no significance was recorded in terms of differences in mouth opening (pair 19) despite one response of trismus when there was no needle change.

-Delayed Scores:

Of the 21 participants, all completed the questionnaires 48-72hrs after their clinical appointments in the no needle change group while a single respondent had missing data and was part of the changed needle group. VASs’ for pain ranged between 0-73 and 0-68 for no needle change (with a mean score of 18.80) and changed needle (mean score of 15.10) groups, respectively. One of the 20 responses had restricted jaw opening less than 3 fingers only in the changed needle group while the remaining 19 subjects reported unrestricted jaw opening.

Analysis using both single and two-tailed paired t-tests was unable to show any significance between pairs relating to either the VAS as well as among the 10 sensory question scores for either the changed or no-needle change (NoCh-Ch) groups ( Table 2). The sensory pain descriptors; shooting, cramping and hot/burning together with emotional descriptors pessimism, guilty, frustration, confidence and recognize were excluded from  Table 2 as paired mean scores were identical & t could not be computed. Additionally, no significance was recorded in terms of differences in mouth opening (pair 16) with 1 of the 20 responses reporting trismus with a needle change.

## Discussion

There were 2 main arms to this study, one which measured pain immediately following 2 needle insertions either with or without a needle change and another that measured the same pain parameters at a second delayed timepoint.

The split mouth design is commonly used in dental clinical trials (10) and was preferred over more traditional parallel arm trials given that the study variable was pain which is very subjective and lends itself to participants acting as self-controls and thereby minimising variation. Additionally, this type of design usually requires half as many participants to achieve the same power compared to conventional parallel arm clinical trials (11). Furthermore, although split mouth design studies have been criticised because of the risk of carry over effects (12), the adequate period between the participants 2 clinical visits would minimise pain transfer between the 2 timepoints.

During the self-screening process, potential participants with systemic condition or those taking any medications that may have influenced sensory pain perception in the orofacial region did not take part in the study (13). This was broadened to include any pulpitis, periodontal disease, periapical disease, cysts, oral lesions or wounds, trigeminal neuralgia, temporomandibular joint disorders (14), as well as dental anxiety and needle phobia associated with a history of vasovagal/collapse events (4) along with bleeding disorders (15). It should be noted that given the high levels of dental knowledge among participants, the absence of pathology was not further verified through comprehensive examination or imaging.

The practice of changing needles between subsequent dental IANBs because of needle tip barbing, has been advocated for reducing pain and tissue trauma which may cause trismus (3). Contrarily, unnecessary changes of needles may add to the risk of needle stick injury as well additional costs and environmental implications due to increased waste generation (16).

Complications such as pain and trismus following IANBs have been widely reported in the literature and the latter mostly attributed to mucosal tearing which may occur during both needle insertion and withdrawal (1,17). Normal mouth opening is usually within the range of 35mm and 45mm as reported by the previous author and can vary depending on age, gender, body size and race (18). Considering these variations, it has been suggested that an accurate method of establishing the difference between normal and reduced mouth opening (trismus) is to use a person’s middle 3 fingers as a reliable measuring tool (18). This was deemed relevant to this study with less than 3 fingers width constituting some level of trismus, especially given participants were all from a dental school and would be familiar with such an approach.

The incidence of trismus as a complication, has been reported at 4.6% (19) and is more likely to appear as a delayed complication one or more days after IANB injections due to inflammation (20). This was the rationale for measuring pain and any restriction in mouth opening both immediately following the needle insertion/withdrawal as well as several days later, with and without, needle change.

The VAS method used in this study, has been widely used to measure dental pain and more specifically during IANB injections (21). The same author reported a mean pain score of 37.1 using the VAS whilst injecting anaesthetic solution. This value is similar to the same day mean VAS pain scores obtained in this study which were 33.05 when there was no needle change and 38.62 with a change of needle. It is however interesting to note comparable scores were obtained in our study despite the fact that there was no anaesthetic fluid injected unlike the referenced study.

Other authors have used VAS’s to measure pain difference during insertion, placement and deposition of anaesthetic during IANB delivery. One study (22) found no statistical significance in pain measurement among any of these 3 moments. On the other hand, other authors showed pain during needle penetration was higher than in the other 2 moments (23), while another study found pain to increase with more rapid injection delivery (24). Additionally, all these studies were conducted with patients who were experiencing various levels of realistic preoperative dental pain. Such variability in results would suggest that direct comparisons of VAS’s when measuring dental pain is difficult given the many variables. This may reflect on the clinical relevance of the current study given that although the presence of any predisposing pain was an exclusion, the failure to find any pain difference with or without a needle change, may be partially due to not collecting data at several moments during the injection. Additional confounders include the fact that our study was conducted without anaesthetic as well as the absence of real life preoperative pain normally associated with most dental visits where an IANB injection is required.

The rationale for not using anaesthetic was to exclude the pain altering effect of the anaesthetic solution as a confounder, given the study’s primary aim was specifically targeting whether the needle change made any difference to the pain. Additionally, fluid pressure associated during dental anaesthetic injections has also been reported as contributing to pain levels (25). This could however be considered a limitation to this study as it did not entirely mimic IANBs in clinical practice.

 The lower delayed VAS scores (15.1 with and 18.8 without needle change), which were approximately half the values of the same day pain scores, could be largely explained by the fact that with the passing of time, substantial pain subsidence could have occurred as well as been affected by a reduced recollection of pain.

Pain is a very complicated construct, suggested as having several dimensions including sensory and emotional which may be difficult to separate (26). This study used a modified McGill Pain Questionnaire (27) with 10 sensory descriptors, namely, throbbing, shooting, sharp, cramping, gnawing, hot/burning, aching, heavy, tender and splitting. Emotional pain was measured using a modified version of the Bodily and Emotional pErception of Pain Questionnaire (28). The latter is commonly referred to as the BEEP questionnaire and the following 12 descriptors of emotional pain were surveyed; irritability, powerlessness, depression, injustice, pessimism, anxiety, guilt, frustration, confidence, recovery, confusion and recognition. The full BEEP questionnaire in its entirety, apart from emotional pain, also includes the pain dimensions of limitations of pain to daily life and interference with personal and social functions. Both of these dimensions were not deemed as relevant to this study and are more suited to chronic pain measurement.

Same day mean scores only showed significance for the sensory pain descriptor “sharp”, with higher values when needle change occurred. This result was not attained in the delayed scores for this descriptor, therefore making any inferences difficult to support. Similarly, a total of 5 of 10 sensory descriptors, had same day higher mean pain scores following a needle change. Although this result may seem counterintuitive, it should be noted that mean scores for 9 of the 10 descriptors, with or without needle change, were all within the mild pain levels with only the descriptor, “sharp”, reaching the moderate pain level. Similarly, with the absence of significance in any of the 10 delayed sensory pain scores, there is insufficient evidence to suggest that needle change reduces sensory pain perception.

There was also a failure to demonstrate significance when using the VAS to measure dental pain, with or without needle change, among both the same day and delayed mean pain scores. These results were additionally mirrored, in the paired emotional pain descriptor mean scores which were very similar both at the same day as well as the delayed scores. On the scale of pain severity ranging from 0-5, same day mean scores ranged from a value of 1.0-1.52 without needle change and 1.0-1.71 with a change in needles. Delayed mean scores ranged from 1.0-1.5 without needle change and 1.0-1.25 with needle change. As mentioned in the results, several descriptors had identical scores of 1.0, both with or without needle change and all the emotional pain descriptor scores lay within a narrow response band (1 to <2), hence the inability to demonstrate significance between paired mean scores. These findings strongly support the premise that changing of needles may not be warranted between subsequent IANBs.

A possible explanation for the similarities in IANB pain score at both time points, with or without a needle change, may lie in the fact that IANBs are given with very small diameter dental needles, hence, any barbing or blunting of the tip does not appear to clinically affect pain. Similarly, another study reported insignificant differences in mean pain scores in abdominal local anaesthetic injections for 21, 23 and 27 gauge needles as measured using a VAS (29). Future research could build on our study using various brand/gauge needles to either confirm or disprove this explanation.

The responses (Yes or No) relating to the ability to fit 3 fingers between the front incisor teeth were overwhelmingly skewed towards a yes response, thereby suggesting an absence of trismus in all but 2 responses. One of these 2 reported some level of trismus, on the same day and without needle change, while the other was in the delayed response following a needle change. This represents a 2.4% incidence of trismus which is well below the 4.6% reported elsewhere (20), however, this may not be accurate given that it could be the same participant that had trismus in the same day scores without a needle change that then had a needle change in the delayed scores.

In summary, self-reported pain after 2 needle insertions during IANB delivery, yielded insignificant mean score differences irrespective of whether there was a needle change. Additionally, pain scores obtained using a variety of measures including a VAS together with sensory and emotional pain questionnaires, were unable to demonstrate significance within the 2 arms.

The findings of this study may cast an element of doubt on previous dental evidence supporting a change in needle between subsequent IANBs.

The study design presented several strengths. The effect of confounders was mitigated during the recruitment phase as this could affect pain response. Additionally, by blinding both participant and clinician as well as incorporating mouth quadrant and needle change randomisation, the effects of bias were reduced. Furthermore, by using a cross- over mouth design, the study participants acted as their own controls and multiple pain measures were utilised across two time points.

Apart from the strengths in the study design there were also several limitations. Modified versions of both the McGill and BEEP questionnaires may have affected the validity of the survey instrument. Additionally, despite the power calculation, the relatively small number of participants used to capture small differences, could have affected the strength of results. Similarly, the relatively homogeneous cohort of dental students and select educators could have potentially biased the results and may limit the generalisability of findings. Furthermore, there was no systematic clinical confirmation of the absence of any pathology beyond participant self- screening. Perhaps the greatest limitation in the study design was the decision to exclude the injection of anaesthetic during the IANB’s. Although the rationale for this has been described earlier, it nonetheless deviates from normal clinical practice.

## Conclusions

The results of this study confirmed the null hypothesis that there was no difference in terms of self-reported levels of pain and trismus, with or without, a needle change between 2 successive IANB injections. Furthermore, a change of needle together with the inherent risk of needle stick injury and waste generation, may not be justified clinically between subsequent IANB injections, despite previous studies justification of needle tip blunting and barbing.

## Figures and Tables

**Table 1:** Same day scores for the VAS (pair 1), sensory (pairs 2-11) and emotional (pairs 12-18) pain descriptors and mouth opening (pair 19).

		Paired sample t-Test		
Pair	Measure (NoCh-Ch)	Mean Difference	t	p (Two-Sided)
1	VAS	-5.571	-1.04	0.311
2	Throbbing	-0.19	-1.073	0.296
3	Shooting	-0.19	-0.89	0.384
4	Sharp	-0.476	-2.911	0.009
5	Cramping	0.143	0.719	0.48
6	Gnawing	-0.143	-1.142	0.267
7	Hot/Burning	-0.143	-1.369	0.186
8	Aching	0.095	0.491	0.629
9	Heavy	0.048	0.37	0.715
10	Tender	0.19	1	0.329
11	Splitting	-0.143	-1.369	0.186
12	Irritability	-0.095	-0.698	0.493
13	Depression	-0.048	-1	0.329
14	Injustice	-0.095	-1.451	0.162
15	Pessimism	0.095	1	0.329
16	Anxiety	-0.19	-1.164	0.258
17	Frustration	-0.095	-1	0.329
18	Recovery	0.286	1.24	0.229
19	Mouth Opening	-0.048	-1	0.329

VAS- visual analogue scale. NoCh-Ch- no change versus change of needle

**Table 2:** Delayed scores for the VAS (pair 1), sensory (pairs 2-8) and emotional (pairs 9-15) pain descriptors and mouth opening (pair 16).

		Paired sample t-Test		
Pair	Measure (NoCh-Ch)	Mean Difference	t	p (Two-Sided)
1	VAS	3.700	0.598	0.557
2	Throbbing	-0.100	-.809	0.428
3	Sharp	0.150	1.371	0.186
4	Gnawing	-0.050	-1.000	0.330
5	Aching	-0.200	-1.453	0.163
6	Heavy	-0.050	-1.000	0.330
7	Tender	-0.150	-1.143	0.267
8	Splitting	0.050	1.000	0.330
9	Irritability	0.100	0.623	0.541
10	Powerless	0.050	0.567	0.577
11	Depression	0.050	1.000	0.330
12	Injustice	0.050	-1.000	0.330
13	Anxiety	0.300	1.674	0.110
14	Recovery	0.050	1.000	0.330
15	Confusion	-0,050	-1.000	0.330
16	Mouth Opening	0.050	1.000	0.330

VAS- visual analogue scale. NoCh-Ch- no change versus change of needle.

## Data Availability

The datasets used and/or analyzed during the current study are available from the corresponding author.
